# Genetically Engineered Cell Membrane-Coated Nanodrug for Targeted Treatment of Thyroid Cancer

**DOI:** 10.34133/bmr.0358

**Published:** 2026-05-07

**Authors:** Shaojie Xu, Xingyin Li, Youyun Peng, Xue Yang, Yuhang Su, Hanning Li, Sining Wang, Xin Huang, Xingrui Li, Yaying Du

**Affiliations:** ^1^ Department of Thyroid and Breast Surgery, Tongji Hospital, Tongji Medical College, Huazhong University of Science and Technology, Wuhan, Hubei 430030, China.; ^2^ Union Hospital, Tongji Medical College, Huazhong University of Science and Technology, Wuhan 430022, China.; ^3^ Tongji Medical College, Huazhong University of Science and Technology, Wuhan, Hubei 430030, China.

## Abstract

Thyroid cancer is the most common endocrine malignancy, particularly in patients with radioactive iodine-refractory differentiated thyroid cancer (RAIR-DTC), who have limited treatment options and poor clinical prognosis. In this study, doxorubicin and sorafenib were loaded into the photothermal conversion agent mesoporous polydopamine, forming mPDS. Tumor cell membrane coating engineering resulted in the formation of mPDS@CAR-M, significantly improving tumor targeting. After internalization by tumor cells, the loaded drugs are rapidly released under near-infrared laser irradiation. mPDS@CAR-M effectively inhibits tumor cell proliferation by enhancing oxidative stress, suppressing the PI3K–AKT–mTOR signaling pathway, and inducing cytotoxic autophagy. By activating harmful autophagy, mPDS@CAR-M further inhibits the epithelial–mesenchymal transition process, reducing tumor cell migration capacity. In vivo experiments showed that mPDS@CAR-M significantly reduced tumor volume, with its therapeutic efficacy closely related to the expression level of the targeted surface antigen. Therefore, mPDS@CAR-M demonstrates significant potential in the treatment of RAIR-DTC, providing a novel direction for further clinical exploration.

## Introduction

Thyroid cancer is the most common endocrine malignancy, with a steadily rising global incidence [1]. In patients with locally advanced or metastatic differentiated thyroid cancer (DTC), approximately two-thirds eventually progress to radioactive iodine-refractory DTC (RAIR-DTC), for which therapeutic options remain limited and clinical outcomes are poor, with a 10-year survival rate of only 15% [2]. Although doxorubicin (DOX) and sorafenib (SOR) are Food and Drug Administration-approved agents for the treatment of this disease [3,4], neither drug has intrinsic tumor-targeting delivery capability, and their clinical utility has declined due to resistance and toxicity. Notably, the DOX–SOR combination has shown efficacy in hepatocellular carcinoma [5,6], but its role in thyroid cancer remains unverified.

Cell membrane-coated nanoparticles (CNPs) are fabricated by cloaking nanoparticle cores with cell membranes, thereby endowing them with the surface markers of the source cells. By modulating membrane protein expression, genetically engineered CNPs can reshape their biointerface and enhance functional performance. The use of chimeric antigen receptor T-cell (CAR-T)CNPs for tumor therapy has been validated in hepatocellular carcinoma models [7]. However, the low transfection efficiency of clinical-grade CAR-T cells makes membrane genetic engineering more challenging, thereby limiting their practical application [8]. In contrast, tumor cells are easily transfected and expanded and possess intrinsic homotypic adhesion properties, making them an ideal cellular source for constructing CAR-engineered membranes.

Tumor-specific antigens, which are preferentially expressed on the surface of tumor cells, represent attractive therapeutic targets [9]. The thyroid-stimulating hormone receptor (TSHR) is consistently expressed in the majority of DTCs, including metastatic and radioiodine-refractory lesions [10], while exhibiting highly restricted expression in nonthyroid tissues. Importantly, patients with RAIR-DTC have typically undergone total thyroidectomy prior to systemic treatment, thereby substantially reducing concerns regarding potential on-target effects in normal thyroid tissue. Based on these considerations, TSHR was selected as the targeting receptor for constructing CAR-engineered membrane-coated nanoparticles (CAR-M).

**Fig. 1.**
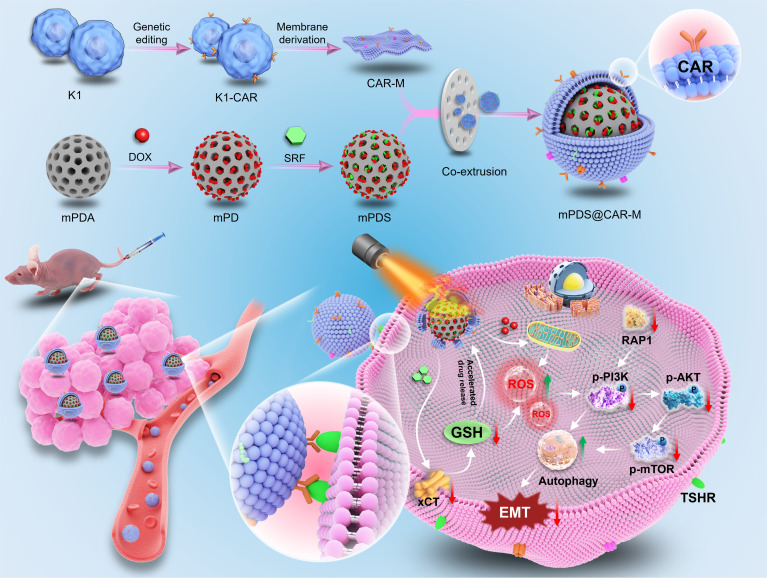
The synthesis and mechanism diagram of mPDS@CAR-M. mPDS@CAR-M targets thyroid cancer by interacting with the thyroid-stimulating hormone receptor (TSHR) on the surface of thyroid cancer cells and promotes cellular-damage-induced autophagy and inhibits epithelial–mesenchymal transition (EMT) through the suppression of the PI3K–AKT–mTOR signaling pathway.

In this study, we developed a genetically engineered, membrane-coated nanodrug (mPDS@CAR-M), in which the photothermal agent mesoporous polydopamine (mPDA) was employed to coload DOX and SOR, followed by cancer cell membrane coating to achieve targeted delivery. Upon near-infrared (NIR) irradiation, the system enabled spatiotemporally controlled drug release through a photothermal effect, thereby achieving a synergistic combination of photothermal therapy, chemotherapy, and targeted therapy for effective tumor ablation. Experimental results demonstrated that mPDS@CAR-M enhanced therapeutic efficacy through autophagy modulation, offering a potential treatment strategy for patients with RAIR-DTC (Fig. 1).

## Materials and Methods

### Establishment of stable cell lines

K1, IHH4, TPC-1, BCPAP, FTC-133, and Nthy-ori 3-1K1 cells were purchased from iCell (Shanghai, China) and cultured in RPMI 1640 medium (Thermo Fisher, Waltham, MA, USA) supplemented with 10% fetal bovine serum (FBS; Thermo Fisher, Waltham, MA, USA). K1 and IHH4 cells were transduced with lentiviruses carrying TSHR or TSHR-specific CAR sequences in serum-free medium. After 12 h, the medium was replaced, and antibiotic selection was applied for 7 to 10 d to establish TSHR- and CAR-expressing stable lines. To generate cell lines with varying levels of TSHR expression, different viral loads were used during transduction. The cells were then sorted or selected based on their TSHR expression levels, as determined by flow cytometry, to obtain populations with low, medium, and high expression of TSHR.

### Flow cytometry analysis

For flow cytometric analysis, cells were harvested and washed with ice-cold phosphate-buffered saline (PBS). Cells were incubated with phycoerythrin (PE) anti-DYKDDDDK Tag Antibody (BioLegend, Cat. No. 637310) and Myc-Tag (9B11) Mouse Monoclonal Antibody (mAb) (Alexa Fluor 647 Conjugate) (Cell Signaling Technology, Cat. No. 2233S) at 4 °C in the dark for the indicated time. For intracellular reactive oxygen species (ROS) detection, cells were incubated with 2′,7′-dichlorodihydrofluorescein diacetate (DCFH-DA; Beyotime, Cat. No. S0035S) according to the manufacturer’s instructions. After staining, cells were washed with PBS, resuspended in PBS, and analyzed on a flow cytometer. Fluorescence signals were acquired, and data were analyzed using FlowJo to quantify fluorescence intensity. At least 10,000 events were recorded per sample, and appropriate controls were used to set compensation and define gating strategies.

### Synthesis and characterization

mPDA (Ruixitech, Cat. No. R-MPDA) was purchased from Xi’an Ruixi Biologicals, while DOX (MedChemExpress, Cat. No. HY-15142A) and SOR (MedChemExpress, Cat. No. HY-10201) were obtained from MedChemExpress. Nanoparticles were prepared by mixing 10 mg of mPDA with 5 mg of DOX, 5 mg of SOR, or both, followed by overnight stirring, centrifugation, washing, and redispersion to obtain mPD, mPS, and mPDS, respectively. Tumor cell membranes were isolated from CAR-K1 and CAR-IHH4 cells using the Minute Plasma Membrane Kit (Invent, Cat. No. SM-005). The extracted tumor cell membranes were sequentially extruded through 400- and 200-nm polycarbonate membranes for approximately 20 cycles to obtain CAR-M. mPDS@M and mPDS@CAR-M were prepared by co-extruding the nanoparticles with tumor cell membranes using the same procedure.

mPDS@CAR-M, CAR-M, and mPDS solutions were sonicated for 5 min, separately applied to copper grids, and negatively stained with uranyl acetate for observation using a low-voltage transmission electron microscope (TEM) system (Hitachi, Japan). mPDS was dropped onto a silicon wafer, dried under an infrared lamp, and then attached to a sample holder with conductive adhesive for scanning electron microscopy imaging (Hitachi, Japan). For cell samples, fixation with osmium tetroxide was followed by PBS washing, dehydration, embedding, sectioning, staining, and air-drying, after which the cellular uptake of nanoparticles could be observed under a TEM.

The particle size distribution and *ζ* potential of mPDA, mPDS, CAR-M, and mPDS@CAR-M solutions were analyzed by dynamic light scattering (Malvern Zetasizer, UK). Serum stability was evaluated by turbidity analysis. After mixing with an equal volume of FBS and incubating at 37 °C, the absorbance at 540 nm was measured at 1, 2, 4, 12, 24, and 48 h using a microplate reader to monitor serum-induced aggregation of nanoparticles. Additionally, hemolysis was evaluated by incubating mouse red blood cell suspensions with PBS (negative control), deionized water (positive control), or serially diluted mPDS@CAR-M solutions for 3 h, followed by centrifugation and measurement of supernatant absorbance at 540 nm. The hemolysis percentage was calculated as follows: Hemolysis (%) = (A_sample − A_negative)/(A_positive − A_negative) × 100%.

Co-extruded cell membrane vesicles and nanoparticles were analyzed by sodium dodecyl sulfate–polyacrylamide gel electrophoresis (SDS-PAGE). Briefly, protein samples were prepared by mixing with loading buffer and then heated at 100 °C for 10 min. The samples were loaded onto a polyacrylamide gel and subjected to electrophoresis. The protein bands were then stained with Coomassie blue.

Powders of mPDS were mixed with KBr and pressed into transparent pellets for recording Fourier transform infrared spectroscopy (FTIR) spectra in the range of 500 to 4,000 cm^−1^ using an FTIR spectrometer (Thermo Fisher, USA). Aqueous solutions of mPD, mPS, and mPDS were placed in quartz cuvettes, and their absorbance at different wavelengths was measured with an ultraviolet–visible (UV–Vis) spectrophotometer (PerkinElmer, USA). Standard curves for DOX and SOR were established by preparing solutions with defined concentration gradients. DOX absorbance was measured with a UV–Vis spectrophotometer, while the peak areas of SOR solutions were determined by high-performance liquid chromatography (HPLC). During mPDS synthesis, the supernatant and wash solutions were collected after 3 washes, and drug contents were quantified using the corresponding calibration curves. The drug-loading efficiency (%) and the drug-loading content (mg/mg) were calculated as follows:

$$\begin{array}{c} \text{Encapsulation efficiency}\;\left(\%\right)\\ =\left(\text{MUsed}-\text{MSupernatant}-\text{MWash}\right)/\text{MUsed}\times 100\% \end{array}$$ (1)

$$\begin{array}{c} \begin{array}{c} \text{Loading efficiency}\;\left(\%\right)=\left(\text{MUsed}-\text{MSupernatant}-\text{MWash}\right)/\\ \left(\text{MAgent}\right)\times 100\% \end{array} \end{array}$$ (2)

where MUsed is the total amount (mg) of the drug initially added; MSupernatant and MWash are the drug amounts (mg) in the supernatant and wash solution, respectively; and MAgent is the weight (mg) of drug-loaded nanoparticles.

The release of drugs was monitored at predetermined time intervals, both with and without NIR irradiation, using the same detection methods. Cumulative release percentage (%) was defined using the following formulas:

$$\begin{array}{c} \text{Cumulative release percentage}\;\left(\%\right)\\ =\text{MReleased}/\text{MLoaded}\times 100\% \end{array}$$ (3)

where MReleased represents the total weight (mg) of the released drug and MLoaded is the total weight (mg) of the initial loaded drug.

For evaluating the photothermal performance of the material, both in vitro and in vivo methods were employed. In vitro, mPDS@CAR-M was irradiated with an 808-nm NIR laser (2 W/cm^2^) in 2-min on/2-min off cycles (4 min per cycle) for 3 cycles, with the temperature monitored by an infrared thermal imager (Testo 865, Testo, Schwarzwald, Germany). In vivo, mPDS@CAR-M was administered to nude mice via tail vein injection; on alternate day, the tumor site was irradiated with laser for 2 min, with local temperature monitored and recorded.

### Cell functional assays

No NIR irradiation was applied in any of the in vitro cell functional experiments unless otherwise specified. K1-TSHR cells were treated with 10 μg/ml of PBS, mPS, mPD, mPDS, mPDS@M, or mPDS@CAR-M for 12, 24, and 48 h or with 5, 10, or 20 μg/ml for 48 h, and cell viability was assessed using the Cell Counting Kit-8 (CCK-8) assay (MedChemExpress, Cat. No. HY-K0301). The live/dead, migration, and colony formation assays used the same treatment groups as the CCK-8 assay. Live/dead staining was performed after treatment with 10 μg/ml for 48 h using the Calcein-AM/Propidium Iodide Live/Dead Staining Kit (Beyotime, Cat. No. C1371S). For migration and colony formation assays, cells were pretreated at 10 μg/ml for 48 h and then processed as follows: for migration, pretreated cells were resuspended in 100 μl of serum-free medium and seeded into the upper chambers of Transwell inserts (8-μm pore size; Corning, USA), with medium containing 10% FBS added to the lower chambers. After 8 to 12 h, migrated cells were fixed and stained. For colony formation, pretreated cells were reseeded at 800 cells per well into 6-well plates, the medium was changed every 3 d, and colonies were fixed, stained, and counted. The same procedures were applied to TSHR-IHH4 cells.

For live/dead staining, K1-TSHR cells were treated with 10 μg/ml of the indicated formulations for 24 h under the following conditions: (a) control, (b) free DOX + free SOR, (c) mPDS@CAR-M, (d) mPDS@CAR-M + NIR, (e) mPDA@CAR-M + NIR, and (f) mPDA@CAR-M + free DOX + free SOR + NIR. The total amounts of DOX and SOR in the free-drug groups were matched to the DOX/SOR payload contained in the 10 μg/ml mPDS@CAR-M dose used in this experiment. NIR irradiation was performed using an 808-nm laser at 2 W/cm^2^, applied in 2-min on/2-min off cycles for 3 cycles, consistent with the parameters described above. The 24-h endpoint was selected to avoid a potential ceiling effect observed at 48 h with 10 μg/ml mPDS@CAR-M, which could mask incremental contributions from NIR irradiation.

K1-TSHR cells were treated with mPDS@CAR-M (0, 2, 5, 10, and 20 μg/ml) for 48 h. ROS levels were measured using DCFH-DA staining followed by flow cytometry. For glutathione (GSH) detection, cell lysates were prepared using Protein Removal Reagent M, followed by freeze–thaw and centrifugation. The absorbance at 412 nm was measured using a microplate reader. Absorbance values for the standards were measured in parallel, and a standard curve was constructed. The total GSH and glutathione disulfide content in the samples were calculated based on the standard curve.

### Immunofluorescence staining

Equal numbers of K1 and K1-TSHR cells were seeded into 24-well plates and treated with 1,1′-dioctadecyl-3,3,3′,3′-tetramethylindodicarbocyanine perchlorate (DiD)-labeled CAR-M. Immunofluorescence (IF) imaging was performed to assess the targeting specificity of CAR-M toward K1 and K1-TSHR cells. In addition, K1-TSHR cells were seeded into 24-well plates and treated with equal amounts of DiD-labeled M and DiD-labeled CAR-M. IF imaging was used to compare the targeting properties of M and CAR-M in K1-TSHR cells. DiD (far-red fluorescent membrane probe; Beyotime, Cat. No. C1039) was used for membrane labeling.

mPDS was labeled with fluorescein isothiocyanate (FITC; green), the membrane was labeled with DiD (red), and the nuclei were stained with 4′,6-diamidino-2-phenylindole (blue). After co-extrusion, dual-fluorescently labeled mPDS@M and mPDS@CAR-M were obtained. K1-TSHR cells were seeded into confocal dishes (3 × 10^4^ cells/well) and cultured for 12 h. Subsequently, 10 μl of mPDS@M or mPDS@CAR-M solution at equal concentrations was added to the cells in FBS-free medium and incubated for 3 h. Images were acquired using confocal laser scanning microscopy (CLSM) with the following excitation/emission parameters: DiD (Ex/Em: 644/663 nm) and FITC (Ex/Em: 492/518 nm).

FITC-labeled mPDS, mPDS@M, and mPDS@CAR-M were intravenously injected via the tail vein into tumor-bearing nude mice. At 24 h postinjection, the biodistribution of nanoparticles was assessed using an in vivo imaging system. After euthanasia, the heart, liver, spleen, lungs, kidneys, and tumor tissues were harvested; rinsed with physiological saline; and imaged for fluorescence using an animal imaging system.

To exclude the ambiguity associated with lipophilic membrane dyes, an extracellular Myc epitope-tagging strategy was used to specifically trace the engineered coating membrane. K1 cells were first transduced with a lentiviral construct encoding a plasma membrane protein carrying an extracellular Myc tag to generate K1-myc cells. K1-myc cells were then further transduced with the TSHR-specific CAR construct to obtain CAR-K1-myc cells. Plasma membranes were isolated from K1-myc and CAR-K1-myc cells using the same membrane extraction procedure described above to generate M-myc and CAR-M-myc vesicles. M-myc or CAR-M-myc vesicles were co-extruded with nanoparticles to prepare Myc-tagged membrane-coated formulations. K1-TSHR and IHH4-TSHR cells were incubated with the corresponding formulations, followed by IF staining using an anti-Myc primary antibody and a Cy3-conjugated secondary antibody. CLSM was performed with the following parameters: Cy3 (Ex/Em: 550/570 nm, red fluorescence).

### Animal models

BALB/c nude mice (male, 3 to 4 weeks) were obtained from Jiangsu GemPharmatech (Jiangsu, China) and maintained under specific-pathogen-free conditions. All procedures were approved by the Animal Ethics Committee of Tongji Hospital, Tongji Medical College (Huazhong University of Science and Technology, Wuhan, China).

To evaluate the therapeutic efficacy of membrane-coated nanoparticles in a K1-TSHR subcutaneous tumor model, male BALB/c nude mice were inoculated subcutaneously with K1-TSHR tumor cells (3 × 10^6^ to 4 × 10^6^ cells in 100 μl). When the tumors reached approximately 6 mm in diameter, the mice were randomly divided into 6 groups: control, mPS, mPD, mPDS, mPDS@M, and mPDS@CAR-M. Different drugs at the same concentration were administered via tail vein injection. At 24 h postinjection, the tumor sites were irradiated with an 808-nm NIR laser (2 W/cm^2^) for 2 min, repeated 3 times. All groups received identical NIR irradiation under the same conditions; therefore, laser exposure was not used as a variable and is not separately indicated in the figures. Tumor volume and body weight were monitored daily. On day 18, all mice were euthanized, and blood was collected via the orbital vein. Tumors, hearts, livers, spleens, lungs, and kidneys were collected. The levels of alanine aminotransferase (ALT), aspartate aminotransferase (AST), creatinine kinase-MB (CK-MB), and lactate dehydrogenase 1 (LDH1) in the blood of nude mice from different groups were measured. Tumors were photographed and weighed, and both tumors and organs were subjected to paraffin embedding, sectioning, and histological analysis. Organ sections were stained with hematoxylin and eosin (H&E). In addition, tumor sections were subjected to H&E staining and immunohistochemistry (IHC) for Ki67 (Ki67 Rabbit mAb, ABclonal, Cat. No. A20018), CD31 (CD31/PECAM1 Rabbit mAb, ABclonal, Cat. No. A19014), LC3B ([KD Validated] LC3B Rabbit mAb, ABclonal, Cat. No. A19665), and phospho-AKT (Ser473) (Phospho-AKT-S473 Rabbit mAb, ABclonal, Cat. No. AP0637).

Further, male BALB/c nude mice were randomly divided into 4 groups for transplantation of different tumor cells (K1-TSHR+, K1-TSHR++, K1-TSHR+++, and wild-type K1). All 4 groups received the same mPDS@CAR-M treatment and NIR irradiation as described above, with drug injections carried out at 3-d intervals between each treatment and tumor size was monitored daily. On day 20, the experiment was terminated, mice were euthanized, and tumors were collected for photographing and weighing.

### RNA sequencing

K1-TSHR cells treated with 10 μg/ml mPDS@CAR-M for 48 h were collected for transcriptome sequencing on the Illumina platform: Total RNA was extracted, complementary DNA (cDNA) libraries were constructed and purified, and RNA sequencing was performed for in-depth analysis. Three biological replicates per group were included.

### Reverse transcriptase quantitative polymerase chain reaction

K1-TSHR cells treated with 10 μg/ml mPDS@CAR-M for 48 h, as well as K1-TSHR cells treated with PBS, were collected. Total RNA was extracted using the RNeasy Mini Kit, and cDNA was synthesized using the iScript cDNA Synthesis Kit. Reverse transcriptase quantitative polymerase chain reaction (PCR) was performed using SYBR Premix Ex Taq and the MxPro Mx3005P real-time PCR detection system (Agilent Technologies, Santa Clara, CA). Primer sequences are listed in Table S1. Glyceraldehyde-3-phosphate dehydrogenase was used as an internal control.

### Western blot

Protein expression levels were assessed by Western blotting and quantified using ImageJ. Briefly, K1-TSHR cells from each group were collected after 48 h of treatment and lysed on ice for 20 min in 1× radioimmunoprecipitation assay buffer supplemented with 1% phenylmethylsulfonyl fluoride. Protein concentrations were determined using a bicinchoninic acid assay kit (Boster, Hubei, China). Lysates were denatured at 100 °C for 10 min, and 20 μg of protein per lane was separated on SDS–PAGE and transferred to polyvinylidene fluoride membranes (Merck Millipore, Germany). Membranes were blocked with a rapid blocking buffer at room temperature for 15 to 30 min, incubated with appropriately diluted primary antibodies overnight at 4 °C on a shaker, washed with tris-buffered saline with Tween-20, and then incubated with species-specific secondary antibodies for 30 to 60 min. Immunoreactive bands were visualized using an enhanced chemiluminescence detection kit (Advansta, USA). Primary antibodies included TSHR (TSHR Antibody, clone 3B12, Santa Cruz), SLC7A11/xCT (Rabbit Polyclonal Antibody [pAb], Cat. No. A13685, ABclonal), Rap1A ([KO Validated] Rabbit pAb, Cat. No. A0975, ABclonal), mTOR ([KD Validated] Rabbit mAb, Cat. No. A25581, ABclonal), phospho-mTOR (Ser2448) (Rabbit mAb, Cat. No. AP0115, ABclonal), PI3K p85α/β (Rabbit PolymAb, Cat. No. A23303PM, ABclonal), phospho-PI3K p85α/p55γ/p85β (Y467/Y464/Y199) (Rabbit pAb, Cat. No. AP0427, ABclonal), pan-AKT (Rabbit mAb, Cat. No. A22412, ABclonal), SQSTM1/p62 (Rabbit mAb, Cat. No. A19700, ABclonal), MMP3 (Rabbit mAb, Cat. No. A11418, ABclonal), N-cadherin ([KO Validated] Rabbit mAb, Cat. No. A19083, ABclonal), and vimentin ([KD Validated] Rabbit mAb, Cat. No. A19607, ABclonal). Goat anti-mouse and goat anti-rabbit secondary antibodies were purchased from Proteintech.

### Statistical analyses

The data were analyzed using *t* tests, one-way analysis of variance or Kruskal–Wallis test in Prism 9 (GraphPad, La Jolla, CA, USA). *P* < 0.05 was considered to indicate significance.

## Results and Discussion

**Fig. 2.**
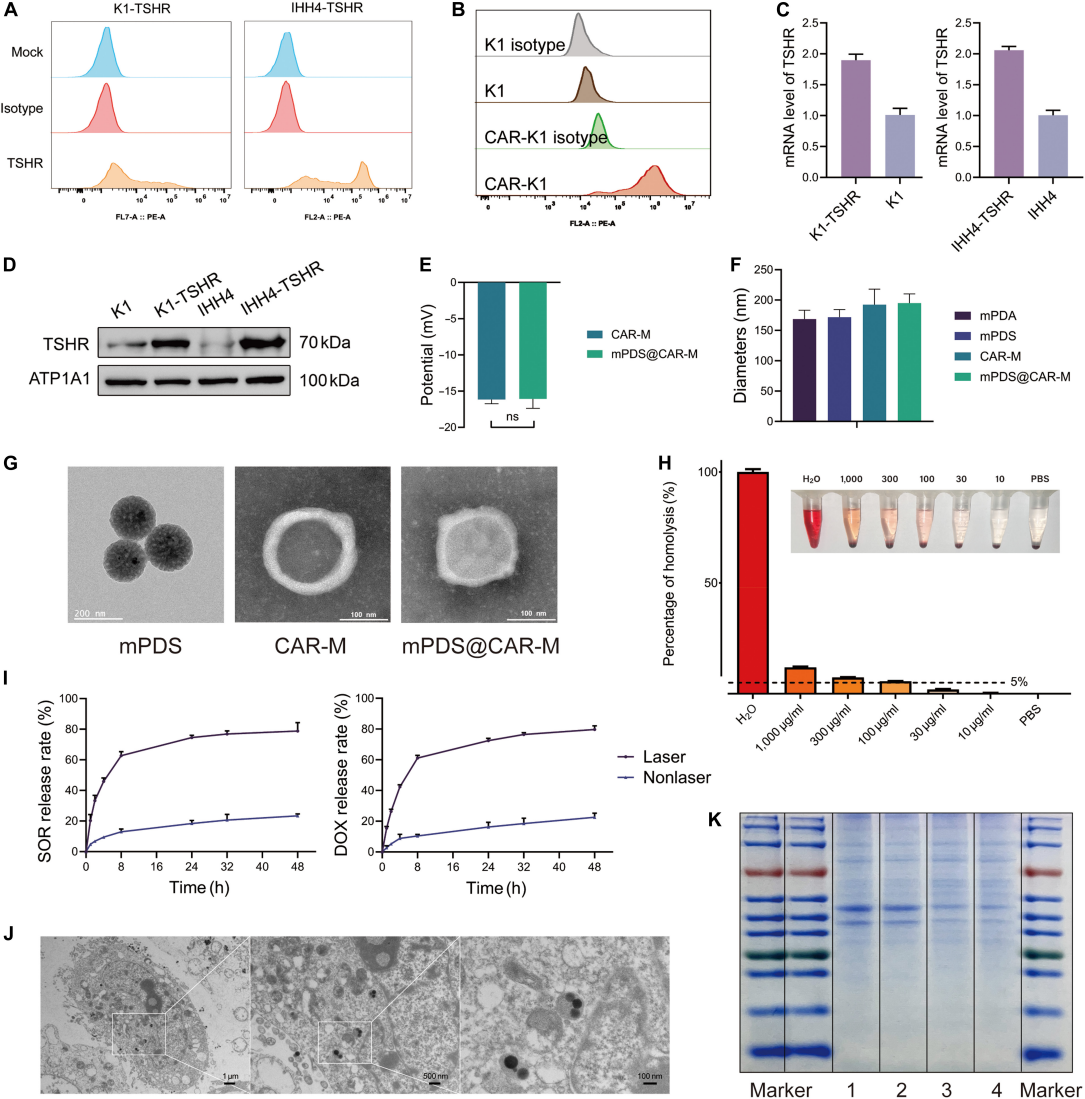
Characterizations of mPDS@CAR-M. (A) Flow cytometry analysis of thyroid-stimulating hormone receptor (TSHR) expression in K1-TSHR and IHH4-TSHR cells. (B) Flow cytometry validation of CAR expression on the surface of CAR-K1 cells. (C) Quantitative polymerase chain reaction (qPCR) analysis confirming TSHR expression in K1-TSHR and IHH4-TSHR cells. (D) Western blot analysis verifying TSHR expression on the cell membranes of K1-TSHR and IHH4-TSHR cells. (E) Zeta potential analysis of CAR-M and mPDS@CAR-M (ns, not significant; *N* = 3). (F) Particle size distribution analysis of mesoporous polydopamine (mPDA), mPDS, CAR-M, and mPDS@CAR-M (*N* = 3). (G) Transmission electron microscope (TEM) images showing the morphology of mPDS, CAR-M, and mPDS@CAR-M (scale bars: 200 and 100 nm). (H) In vitro hemocompatibility evaluation of mPDS@CAR-M. Hemolysis (%) was calculated using positive (water) and negative (phosphate-buffered saline [PBS]) controls. The dashed line indicates the 5% hemolysis safety threshold (*N* = 3). (I) Drug release profiles of doxorubicin (DOX) and sorafenib (SOR) from mPDS@CAR-M under near-infrared (NIR) irradiation and nonirradiated conditions. (J) Cellular uptake of mPDS@CAR-M observed by TEM (scale bars: 1 μm, 500 nm, and 100 nm). (K) Sodium dodecyl sulfate–polyacrylamide gel electrophoresis (SDS-PAGE) analysis of membrane proteins derived from K1 cells (lane 1), mPDS@M (lane 2), CAR-K1 cells (lane 3), and mPDS@CAR-M (lane 4).

The expression of TSHR in different thyroid cancer cell lines remains controversial [10]. We first confirmed that TPC-1, K1, IHH4, BCPAP, and Nthy-ori 3.1 cells completely lacked TSHR expression, whereas FTC-133 cells expressed TSHR (Fig. S1A). To further investigate, we selected the K1 cell line derived from a primary tumor and the IHH4 cell line derived from lymph node metastatic lesions and established 2 stable TSHR-expressing cell models, K1-TSHR and IHH4-TSHR, via lentiviral transduction. TSHR was successfully expressed and localized to the plasma membrane, as confirmed by flow cytometry (Fig. 2A), qPCR (Fig. 2C), and Western blotting (Fig. 2D). The construction of CAR-K1 cells followed a similar procedure to that used for our previously established CAR-T cells [11], and CAR expression on the cell surface was confirmed by flow cytometry (Fig. 2B).

The preparation of mPDS@CAR-M involved 3 sequential steps: (a) isolating plasma membrane vesicles (CAR-M) from CAR-K1 cells after the removal of organelle membranes; (b) loading DOX and SOR into the mesoporous structure of mPDA to construct nanoparticles (mPDS); and (c) mixing CAR-M with mPDS, followed by sequential co-extrusion of the mixture through 400- and 200-nm polycarbonate membranes to encapsulate mPDS within the membrane vesicles.

Dynamic light scattering analysis revealed that mPDS@CAR-M exhibited an average diameter of approximately 190 nm, with the surface potential largely restored to the level of CAR-M (Fig. 2E and F). The surface structure of mPDS was observed through scanning electron microscopy (Fig. S1B), while TEM (Fig. 2G) confirmed the morphological characteristics of mPDS, CAR-M, and mPDS@CAR-M. Additionally, the biological TEM images demonstrated the cellular uptake behavior and the distribution of mPDS@CAR-M within the cells (Fig. 2J). Notably, the obtained mPDS@CAR-M maintained structural stability for at least 2 d (Fig. S1C and F), ensuring the technical feasibility of subsequent experiments.

Successful drug loading was further validated by UV–Vis spectroscopy and HPLC analysis. Overlayed UV–Vis spectra confirmed DOX encapsulation (Fig. S1D), while HPLC chromatograms verified SOR loading (Fig. S1E). Quantitative analysis based on standard calibration curves (Fig. S1G) showed that the drug-loading efficiency and encapsulation efficiency of DOX in mPDS were 15.9% and 37.7%, respectively, whereas those of SOR were 16.2% and 38.8%, respectively. For single-drug formulations, the loading and encapsulation efficiencies were 18.2% and 44.4% for mPD and 19.3% and 47.9% for mPS, respectively (Table S2). A limitation of our study is that we did not investigate the optimal synergistic ratio of DOX and SOR, which could impact therapeutic efficacy. However, due to challenges in drug loading, we adopted this ratio (approximately 1:1) as a reasonable compromise for the current study.

The biocompatibility of mPDS@CAR-M was evaluated at 5 concentrations (10, 30, 100, 300, and 1,000 μg/ml). After background correction, the hemolysis rate remained below 5% at concentrations ≤30 μg/ml, which is generally accepted as the safety threshold for hemocompatibility (Fig. 2H). Furthermore, neither mPDA nor mPDA@CAR-M nor NIR irradiation alone showed any cytotoxic effects (Fig. S1J).

**Fig. 3.**
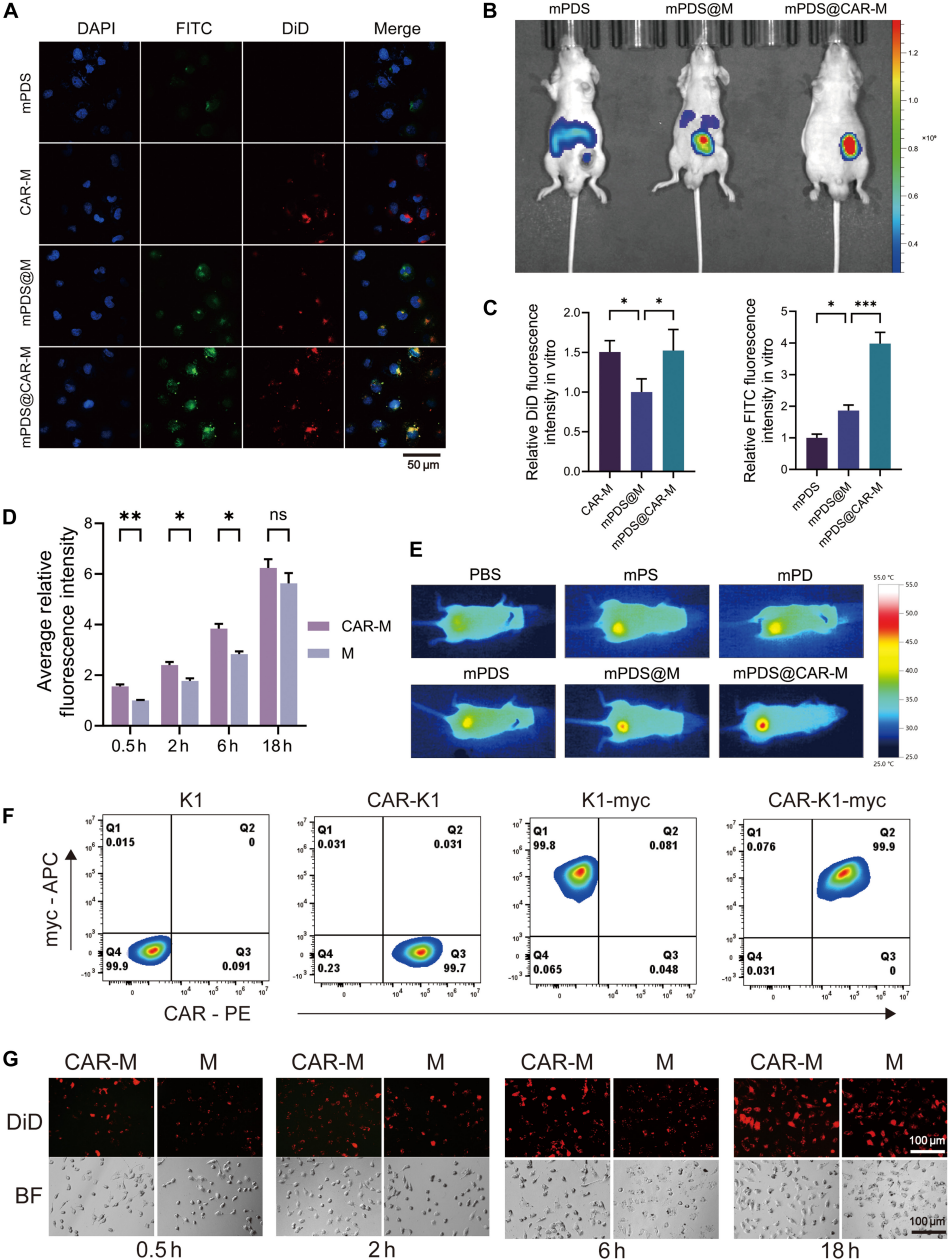
In vitro and in vivo tumor targeting of mPDS@CAR-M. (A) mPDS was labeled with fluorescein isothiocyanate (FITC; green), and the membrane was labeled with 1,1′-dioctadecyl-3,3,3′,3′-tetramethylindodicarbocyanine perchlorate (DiD; red). The tumor cell-targeting ability of mPDS@CAR-M was investigated in vitro (scale bar: 50 μm). (B) The tumor accumulation of mPDS@CAR-M was evaluated using a xenograft tumor model in vivo (**P < 0.01, *N* = 3). (C) Statistical analysis of the fluorescence intensity of DiD and FITC in different groups in vitro (*P < 0.05 and ***P < 0.001, *N* = 3). (E) In vivo photothermal effect under 808-nm near-infrared (NIR) irradiation. All groups were exposed to identical laser parameters (2 W/cm^2^, 2 min). (F) Flow cytometry analysis of K1-myc and CAR-K1-myc cells, showing Myc-tag expression. (G) Binding of CAR-M and M to K1-TSHR cells at different time points (scale bar: 100 μm) and (D) statistical analysis (ns, not significant; *P < 0.05 and **P < 0.01, *N* = 3). TSHR, thyroid-stimulating hormone receptor.

The in vivo photothermal effect forms the theoretical basis of photothermal therapy, in which elevated temperatures of 46 to 52 °C induce microvascular thrombosis, ischemia, and rapid cell death [12]. We first assessed the in vitro photothermal performance of mPDS@CAR-M. Upon 808-nm NIR laser irradiation, the temperature of the mPDS@CAR-M suspension reached 55 °C within 2 min and returned to room temperature approximately 2 min after laser withdrawal, demonstrating reproducible and stable photothermal behavior (Fig. S1H and I). The in vivo photothermal effect of mPDS@CAR-M was further confirmed (Fig. 3E). Irradiation of the PBS group under identical laser parameters resulted in minimal temperature elevation, confirming that the observed heating effect was primarily due to the photothermal conversion capability of mPDS@CAR-M. In the absence of NIR irradiation, the vesicular coating effectively blocked mPDA, thereby restricting drug release from the nanoparticles [13]; the cumulative release of DOX and SOR did not exceed 30% within 48 h. In contrast, NIR irradiation induced vesicle rupture due to localized heating and increased the kinetic energy of drug molecules in solution [14]. The cumulative release of both drugs increased markedly within the first 8 h and plateaued thereafter at approximately 80% (Fig. 2I).

SDS-PAGE revealed that mPDS@M and mPDS@CAR-M shared nearly identical membrane protein compositions with their corresponding vesicles (Fig. 2K). Conserved adhesion molecules facilitated recognition and homotypic targeting of tumor cells through the formation of adhesive junctions [15,16], while CD47 functioned as a “self-marker” to protect against macrophage-mediated phagocytosis [17].

We evaluated the tumor-targeting ability of mPDS@CAR-M both in vitro and in vivo. mPDS was labeled with FITC (green), while the cell membrane was labeled with DiD (red). After coincubating K1-TSHR cells with mPDS, CAR-M, mPDS@M, or mPDS@CAR-M, drug uptake was assessed by confocal microscopy. The mPDS@CAR-M group exhibited the strongest green fluorescence signal, with no significant difference in red fluorescence compared to the CAR-M group (Fig. 3A and C), indicating that the in vitro tumor-targeting capability of mPDS@CAR-M was entirely derived from CAR-M. The tumor accumulation capacity of nanodrugs was further investigated in vivo using a xenograft tumor model and FITC-labeled mPDS. Nude mice were intravenously injected with mPDS, mPDS@M, or mPDS@CAR-M, and the fluorescence intensity was measured 24 h later using an in vivo imaging system. The fluorescence intensity at the tumor site in the mPDS@CAR-M group was significantly higher than those in the other groups (Fig. 3B and Fig. S2E). Meanwhile, the accumulation in the liver, spleen, and kidneys was significantly lower than that in the other groups (Fig. S2A and B). This confirms that mPDS@CAR-M exhibits effective tumor-targeting ability in vivo, which is attributed to the enhanced permeability and retention effect of the nanoparticles and the active targeting capability conferred by the CAR-engineered membrane (CAR-M).

Since DiD is a nonspecific membrane dye, we further validated the coating membrane signal using an extracellular Myc epitope tag, which is present only on the membranes used for nanoparticle coating (Fig. 3F). CAR-M-myc-coated nanoparticles exhibited significantly stronger Myc/Cy3 signals than M-myc-coated nanoparticles in both K1-TSHR and IHH4-TSHR cells, confirming that the enhanced targeting is not an artifact of DiD labeling (Fig. S2F and G). Notably, K1-derived membranes showed detectable association with IHH4-TSHR cells, indicating that membrane-mediated interactions are not strictly limited to the same tumor cell line. However, CAR-M-myc consistently produced stronger signals than M-myc, demonstrating that CAR recognition further enhances targeting beyond intrinsic membrane interactions.

We aimed to evaluate the relationship between the targeting ability of CAR-M and time. K1-TSHR cells were treated with DiD-labeled M or CAR-M. At 0.5 h posttreatment, a higher signal of CAR-M was detected on the cell membrane of K1-TSHR cells compared to that on the M group (Fig. 3D and G). This increased adhesion capability persisted up to 6 h posttreatment. At 18 h, no significant difference in fluorescence intensity was observed between the CM-DiD and CAR-M-DiD groups. The specific affinity of CAR-M was further evaluated in vitro by co-culturing with K1-TSHR and wild-type K1 cells (Fig. S2C and D). At different time points, K1-TSHR cells showed higher uptake efficiency and stronger fluorescence intensity compared to K1 cells.

**Fig. 4.**
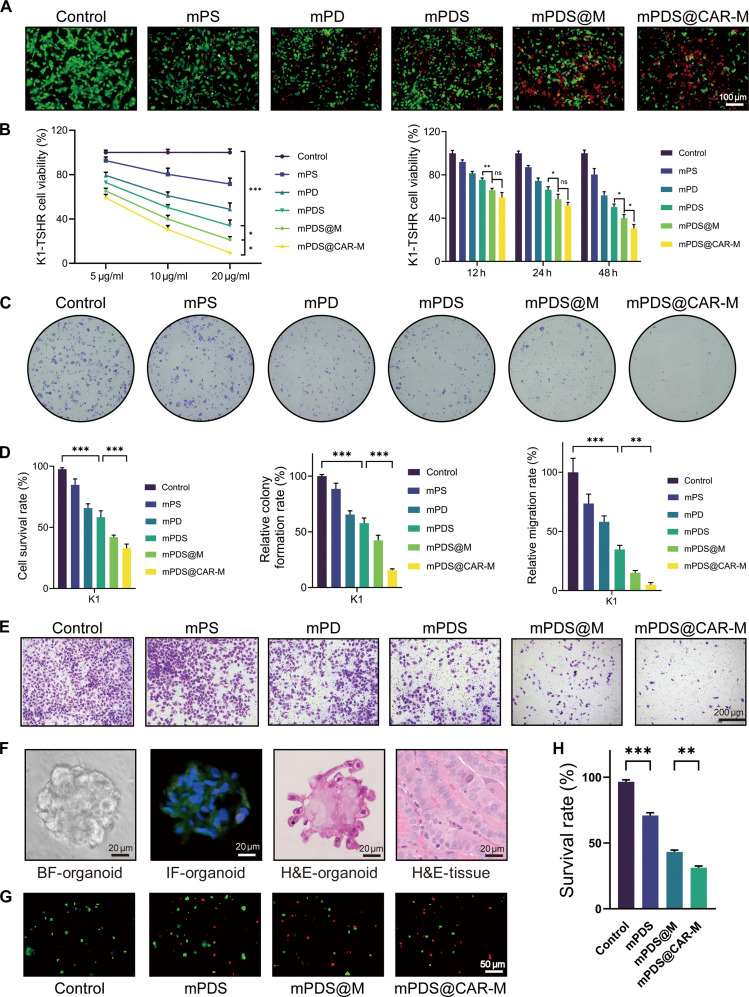
Antitumor efficacy of mPDS@CAR-M in vitro. All cell functional assays and organoid experiments were performed without near-infrared (NIR) irradiation, unless otherwise specified. (A) Live/dead staining assay demonstrating the effect of mPDS@CAR-M on K1-TSHR cells’ death (scale bar: 100 μm). TSHR, thyroid-stimulating hormone receptor. (B) Cell Counting Kit-8 (CCK-8) assay assessing the effect of mPDS@CAR-M on K1-TSHR cells’ viability (ns, not significant; *P < 0.05, **P < 0.01, and ***P < 0.001, *N* = 3). (C) Colony formation assay showing the impact of mPDS@CAR-M on the colony formation of K1-TSHR cells. (D) Statistical analysis of live/dead staining, colony formation, and Transwell assays (**P < 0.01 and ***P < 0.001, *N* = 3). (E) Transwell assay evaluating the effect of mPDS@CAR-M on K1-TSHR cells’ migration (scale bar: 200 μm). (F) Light microscopy images of thyroid cancer organoids, hematoxylin and eosin (H&E) staining, immunofluorescence, and corresponding H&E-stained tumor tissue sections (scale bar: 20 μm). (G) Antitumor effect of mPDS@CAR-M in thyroid cancer organoids and (H) quantitative analysis (**P < 0.01 and ***P < 0.001, *N* = 3).

We next investigated the antitumor effects of mPDS@CAR-M in thyroid cancer cells. Tumor cells were incubated with different concentrations of PBS, mPS, mPD, mPDS, mPDS@M, or mPDS@CAR-M for various durations. CCK-8 assays demonstrated that compared with mPDS@M, mPDS@CAR-M further reduced cell viability after 48 h of treatment (Fig. 4B), exhibiting both time- and concentration-dependent cytotoxicity. Based on these findings, 48 h and 10 μg were selected as the parameters for subsequent functional assays. Live/dead staining (Fig. 4A and D), colony formation assays (Fig. 4C and D), and Transwell assays (Fig. 4E and D) collectively demonstrated the superiority of mPDS@CAR-M in terms of cytotoxicity, inhibition of colony formation, and suppression of cell migration, respectively, confirming its potent in vitro antitumor activity. These findings were further validated in IHH4-TSHR cells, yielding consistent results (Fig. S3A to E). The observed cytotoxicity is attributed to the tumor cell membrane-coated nanomaterials, which facilitate the entry of the nanoparticle core into tumor cells via membrane fusion. The CAR structure cross-links with TSHR, enhancing the adhesion of the nanocarrier to the tumor. Additionally, the acidic microenvironment weakens the π–π and electrostatic interactions within the mPDA system, thereby promoting drug release.

To further dissect the respective contributions of chemotherapy and photothermal therapy under matched-dose conditions, we compared different treatment combinations in a unified live/dead assay. Free DOX + SOR and mPDA@CAR-M + NIR each induced moderate cytotoxicity, reflecting the independent contributions of chemotherapy and photothermal treatment. mPDS@CAR-M + NIR exhibited greater cytotoxicity compared with mPDS@CAR-M alone, indicating that NIR irradiation enhances the therapeutic efficacy of the drug-loaded nanoplatform. Although this enhancement may represent a complementary effect rather than a strictly synergistic interaction between photothermal therapy and chemotherapy, it nevertheless supports the added value of NIR activation in this system.

Subsequently, we assessed the therapeutic potential of nanomedicines in organoid models derived from thyroid cancer patients. Based on previous reports [18], thyroid cancer organoid systems were successfully established from tumors surgically resected from patients and passaged once the average size reached 100 μm (Fig. S4A). H&E staining was used to compare the histological features of the organoids with those of the corresponding primary tumors. IF staining revealed that the expression of TSHR (green) was well preserved in the organoids, while the expression of Ki67 (red) was relatively weak (Fig. 4F). Consistent with the cell assays, treatment with nanoparticle drugs showed that the mPDS@CAR-M group exhibited the strongest antitumor effect (Fig. 4G and H). Additionally, the morphological changes of the organoids before and after treatment were shown under optical microscopy (Fig. S4B).

**Fig. 5.**
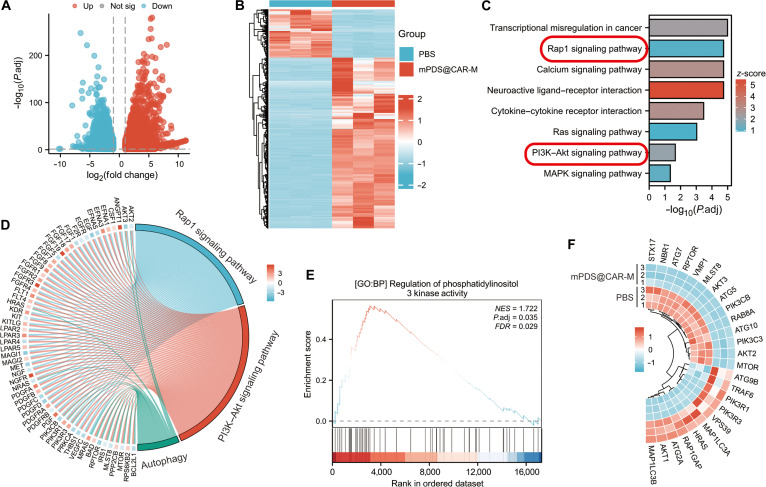
Transcriptome analysis of K1-TSHR cells treated with mPDS@CAR-M. TSHR, thyroid-stimulating hormone receptor. (A and B) Volcano plot and heatmap of differentially expressed genes (DEGs) in K1-TSHR cells between the PBS group and the mPDS@CAR-M group. (C) Gene Ontology (GO) and Kyoto Encyclopedia of Genes and Genomes (KEGG) enrichment analysis of DEGs in K1-TSHR cells. (D) KEGG and logFC-based combined enrichment analysis revealing significant enrichment of autophagy-related pathways. (E) Gene set enrichment analysis (GSEA) identifying enrichment of PI3K activity-related pathways. (F) Heatmap of key genes in the RAP1–PI3K–autophagy pathway.

We further investigated the potential mechanisms underlying the inhibitory effects of mPDS@CAR-M on tumor growth and metastasis. RNA sequencing was performed on the K1-TSHR cell line from the control group (PBS) and the mPDS@CAR-M group immediately after treatment. The alignment rate of each sample to the reference genome exceeded 95% (Table S3). Differential gene expression analysis was performed using the DESeq2 algorithm, with a screening threshold of *P*-adjust <0.05 and a fold change ≥2. A total of 3,274 differentially expressed genes were identified between the control and mPDS@CAR-M groups, comprising 2,227 up-regulated and 1,047 down-regulated genes (Fig. 5A). A binary heatmap visualized these transcriptomic differences, with up-regulated genes in red and down-regulated in blue (Fig. 5B). Kyoto Encyclopedia of Genes and Genomes (KEGG) pathway analysis revealed that the RAP1 signaling pathway and the PI3K–AKT signaling pathway were among the most significantly enriched pathways (Fig. 5C). The RAP1 signaling pathway activates the PI3K–AKT pathway, thereby promoting the generation of its downstream effects [19]. Gene set enrichment analysis similarly highlighted the enrichment of pathways associated with the regulation of PI3K activity (Fig. 5E). Genetic alterations in the PI3K–AKT signaling pathway constitute a critical component of the pathogenesis of thyroid cancer and are fundamental driving factors in the development of RAIR-DTC [20]. Furthermore, combined KEGG and logFC enrichment analysis demonstrated significant enrichment of autophagy-related pathways (Fig. 5D). mTOR, located downstream of the PI3K/AKT signaling pathway, is closely associated with the regulation of autophagy through its phosphorylation [21,22]. A circular heatmap illustrates the expression of key genes in the RAP1–PI3K–autophagy pathway (Fig. 5F).

**Fig. 6.**
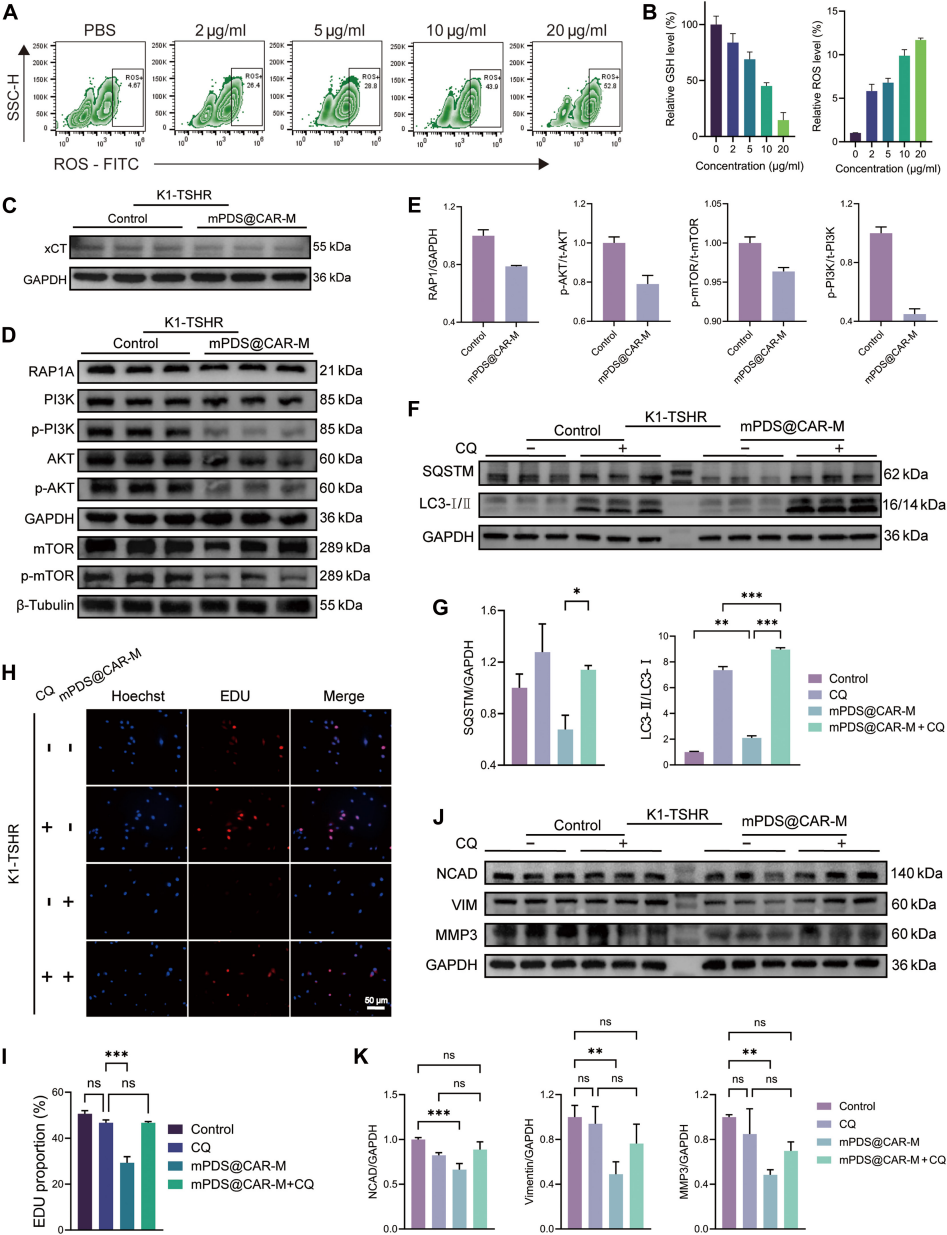
The potential mechanisms of mPDS@CAR-M therapy. (A) Flow cytometry analysis of intracellular reactive oxygen species (ROS) levels in K1-TSHR cells treated with different concentrations of mPDS@CAR-M. TSHR, thyroid-stimulating hormone receptor. (B) Quantitative analysis of the relationship between intracellular ROS and glutathione (GSH) levels and mPDS@CAR-M concentration (*n* = 3, mean ± SD). (C) Western blot analysis verifying the inhibitory effect of mPDS@CAR-M on xCT expression in K1-TSHR cells. (D) Western blot analysis of key RAP1–PI3K–AKT–mTOR pathway proteins in K1-TSHR cells following mPDS@CAR-M treatment. (E) Quantitative analysis of the proteins in (D) (*n* = 3, mean ± SD). (F) Western blot analysis of autophagy-related protein expression in K1-TSHR cells after mPDS@CAR-M and chloroquine (CQ) treatment. (G) Quantitative analysis of the proteins in (F) (*n* = 3, mean ± SD, *P < 0.05, **P < 0.01, ***P < 0.001). (H) 5-Ethynyl-2′-deoxyuridine (EDU) assay showing the reversal of mPDS@CAR-M’s antitumor effects by CQ on K1-TSHR cell proliferation (scale bar: 50 μm). (I) Quantitative analysis of the EDU assay results (*n* = 3, mean ± SD; ns, not significant; ***P < 0.001). (J) Western blot analysis of epithelial–mesenchymal transition (EMT)-related protein expression in K1-TSHR cells after mPDS@CAR-M and CQ treatment. (K) Quantitative analysis of the proteins in (J) (*n* = 3, mean ± SD, **P < 0.01 and ***P < 0.001).

Intracellular GSH and ROS are essential regulators of redox homeostasis, as well as critical determinants of cell proliferation and death [23]. As a major intracellular antioxidant, GSH plays a central role in neutralizing ROS [24]. Studies have shown that polydopamine itself can lead to GSH depletion, possibly due to the antioxidant GSH promoting the depolymerization and degradation of mPDA [25]. Depletion of GSH results in excessive ROS accumulation, which induces oxidative stress, causes cellular damage, and disrupts key signaling pathways [26]. We observed that mPDS@CAR-M induced GSH depletion and ROS accumulation in a concentration-dependent manner (Fig. 6A and B). GSH levels in tumor cells typically reach up to 10 mM, and it can synergistically accelerate the release of drug-loaded mPDA in the acidic microenvironment of tumor cells [27,28]. The released SOR, an xCT inhibitor, inhibits the activity of the SLC7A11 transporter (xCT), thereby suppressing the intracellular transport of GSH and exacerbating GSH depletion. We further validated the inhibitory effect of mPDS@CAR-M on xCT (Fig. 6C). Meanwhile, the released DOX accumulates in the mitochondria, disrupting the respiratory chain and ultimately elevating ROS levels [29].

We next examined key regulators of the RAP1–PI3K–AKT–mTOR signaling pathway by qPCR. Up-regulation of RAP1GAP facilitated the conversion of RAP1-GTP to RAP1-GDP, thereby suppressed RAP1 signaling [30]. Reduced RAP1 activity in turn diminished its ability to recruit and activate PI3K, weakening the upstream signaling drive [31,32]. Down-regulation of PIK3C3, PIK3CB, AKT1, AKT2, AKT3, MTOR, RPTOR, and MLST8 further suggested inhibition of the PI3K–AKT–mTOR pathway (Fig. S5A). Consistently, the protein expression levels of p-PI3K, p-AKT, and p-mTOR were significantly lower in the mPDS@CAR-M group than in the control group (Fig. 6D and E).

As a canonical regulatory axis of autophagy, inhibition of mTOR removes the brake on autophagy [33]. qPCR analysis revealed that the messenger RNA (mRNA) levels of autophagy-related genes MAP1LC3A and MAP1LC3B were significantly increased in the mPDS@CAR-M group compared with those in the control group (Fig. S5B), indicating the activation of autophagy at the transcriptional level. Western blot analysis further confirmed this finding, showing a marked increase in the LC3-II/LC3-I ratio. Upon treatment with the autophagy inhibitor chloroquine (CQ), which blocks the fusion of autophagosomes with lysosomes, LC3-II accumulated further, suggesting enhanced autophagic flux (Fig. 6F and G). Moreover, rescue experiments demonstrated that CQ completely reversed the antiproliferative effect of mPDS@CAR-M on tumor cells (Fig. 6H and I), indicating that mPDS@CAR-M inhibits tumor cell proliferation by activating cell-damaging autophagy.

We also assessed epithelial–mesenchymal transition (EMT) related markers. The down-regulation of FN1, VIM, CDH2, MMP2, MMP9, and SNAIL mRNA levels, along with the up-regulation of CDH1 mRNA, in the mPDS@CAR-M group compared to the control group (Fig. S5C), along with a significant decrease in the expression of EMT-related proteins NCAD, VIM, and MMP3 (Fig. S5D and E), suggests that mPDS@CAR-M effectively inhibits the EMT process, thereby suppressing tumor metastasis. Furthermore, when autophagy was inhibited by CQ, the expression of EMT-related proteins was restored to levels similar to those in the control group (Fig. 6J and K). This suggests that mPDS@CAR-M inhibits EMT through the activation of detrimental autophagy.

To further validate the proposed mechanism, we performed additional experiments using the IHH4 cell line. Specifically, we repeated the Western blot analysis of key proteins involved in the PI3K–AKT–mTOR signaling pathway (Fig. S5G), autophagy (Fig. S5H), and EMT (Fig. S5I and F), similar to the experiments conducted with the K1 cell line. The results from the IHH4 cell line were consistent with those from K1 cells, confirming that the suppression of the PI3K–AKT–mTOR pathway relieved the inhibition of cell-death-associated autophagy and led to the inhibition of autophagy-induced EMT, which supports the robustness of the proposed mechanism.

**Fig. 7.**
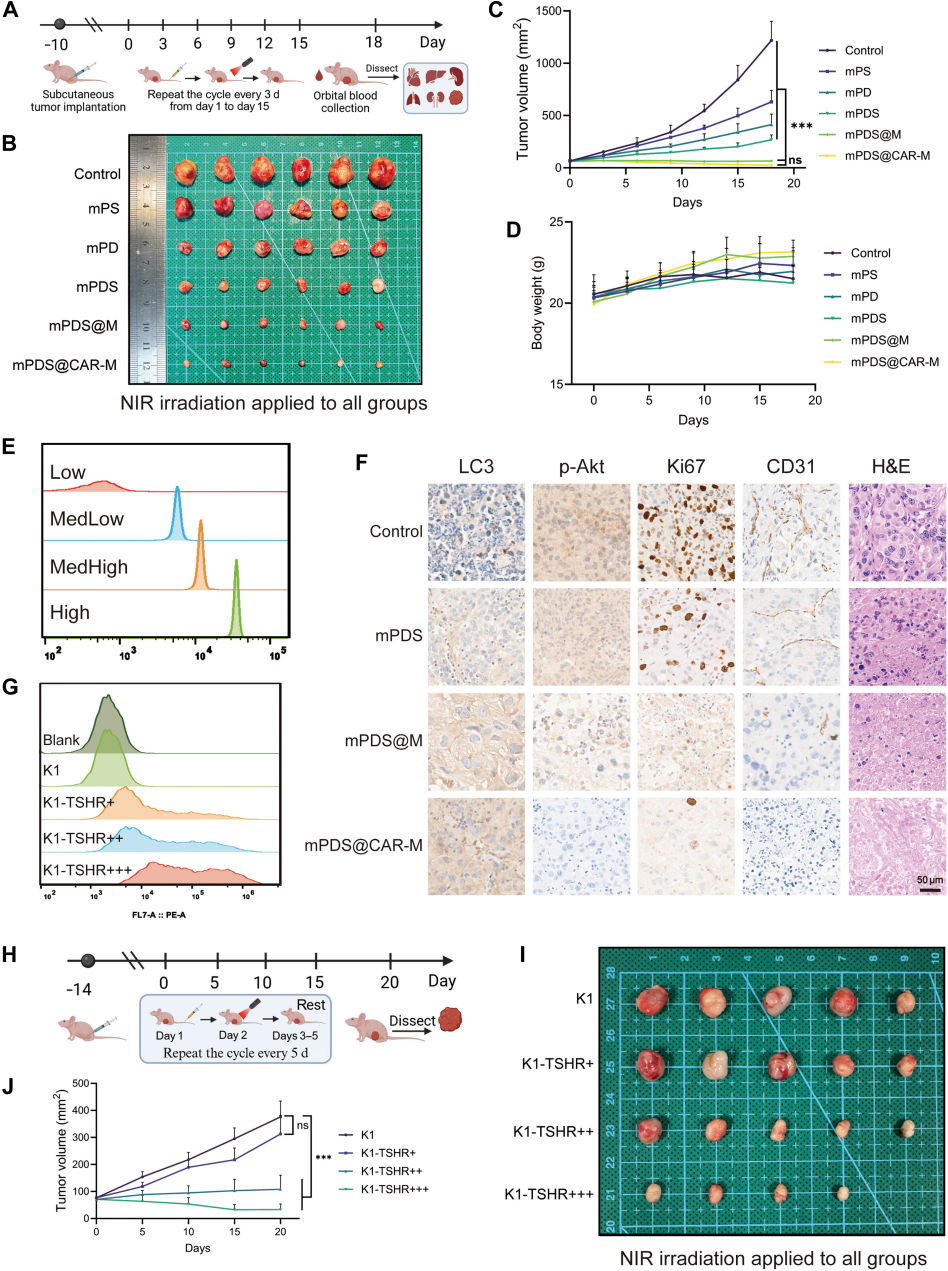
Targeted antitumor therapy of mPDS@CAR-M in vivo. (A) Schematic of the experimental timeline for tumor implantation, nanoparticle injection, laser irradiation, and tumor growth monitoring. (B and C) Nude mice were divided into groups: control, mPS, mPD, mPDS, mPDS@M, and mPDS@CAR-M. Tumor volumes were recorded over time (ns, not significant; ***P < 0.001, *N* = 6). (D) Monitoring of body weight in nude mice across above groups. (E) Standard curve for phycoerythrin (PE) channel fluorescence intensity and the number of PE molecules bound to single cells. (F) Hematoxylin and eosin (H&E) staining and immunohistochemistry (IHC) staining of Ki67, CD31, LC3, and p-AKT in the control, mPDS, mPDS@M, and mPDS@CAR-M groups. (G) Relative quantitative analysis of thyroid-stimulating hormone receptor (TSHR) expression levels on the surface of wild-type K1, K1-TSHR+, K1-TSHR++, and K1-TSHR+++ cells. (H) Experimental timeline for subcutaneous tumor formation in cells with varying TSHR expression levels, followed by mPDS@CAR-M injection, irradiation, and tumor growth monitoring. (I and J) Tumor growth curves and final volumes for subcutaneous tumors of K1, K1-TSHR+, K1-TSHR++, and K1-TSHR+++ cells (ns, not significant; ***P < 0.001, *N* = 5). Created in https://BioRender.com

To evaluate the in vivo antitumor efficacy of mPDS@CAR-M, a subcutaneous xenograft model was established in BALB/c nude mice using K1-TSHR cells. On day 10 after tumor implantation, mice received intravenous injections of the nanoparticle formulations via the tail vein, followed by NIR laser irradiation of the tumor site the next day (Fig. 7A). The results showed that compared to the control and mPDS groups, both the mPDS@M and mPDS@CAR-M groups exhibited significantly reduced tumor volumes (Fig. 7B and C) and tumor weights (Fig. S6B), while no significant differences in body weight were observed among the groups (Fig. 7D). CK-MB and LDH1 are markers of myocardial injury, while AST and ALT serve as indicators of liver function, with ALT being more liver specific and AST reflecting both hepatic and cardiac damage [34,35]. Following treatment, the levels of CK-MB, LDH1, AST, and ALT in the mPDS@M and mPDS@CAR-M groups showed no significant differences compared with those in the control group, indicating favorable biosafety. In contrast, CK-MB and AST levels were significantly elevated in the mPDS group (Fig. S6A). Furthermore, H&E staining revealed no evident signs of cardiac damage, pulmonary toxicity, splenic inflammation, or hepatic and renal injury in the mPDS@M and mPDS@CAR-M groups (Fig. S6D).

H&E staining revealed disorganized tumor cell arrangement, cytoplasmic vacuolization, reduced nucleocytoplasmic contrast, and evidence of necrosis or fibrosis in the mPDS@M and mPDS@CAR-M groups. IHC analysis further demonstrated a marked reduction in Ki67-positive cells, indicating that mPDS@CAR-M exerted stronger antitumor proliferative in vivo. The expression of the neovascularization marker CD31 was significantly reduced in the mPDS@CAR-M group compared to that in the control group, which may be attributed to thermal ablation and the anti-angiogenic effect of SOR [36]. In the mPDS@CAR-M group, the number of p-AKT-positive cells was significantly reduced, while the number of LC3-positive cells was markedly increased. This further confirms that mPDS@CAR-M kills tumor cells through the PI3K–AKT pathway and damage-induced autophagy (Fig. 7F).

Although the antitumor effect of the mPDS@CAR-M group appeared stronger than that of the mPDS@M group (Fig. 7B), no statistically significant differences in final tumor volume or weight were observed between the 2 groups (Fig. 7C and Fig. S6B and C). We speculated that insufficient TSHR expression may have limited the in vivo targeting efficacy of the CAR structure. To investigate this, we generated cell lines with different levels of TSHR expression by transducing K1 cells with lentivirus at varying titers, designated as K1-TSHR+, K1-TSHR++, and K1-TSHR+++. Using calibration beads with 4 levels of PE conjugation, we established a standard curve that converted PE channel fluorescence intensity into the number of PE molecules bound per cell (Fig. 7E). Quantitative analysis indicated that the relative surface expression levels of TSHR in K1-TSHR+, K1-TSHR++, and K1-TSHR+++ were approximately 1:2:6 (Fig. 7G and Table S4).

To evaluate the impact of TSHR expression on therapeutic efficacy, wild-type K1 cells and 3 engineered cell lines with varying TSHR levels were implanted in nude mice and treated with mPDS@CAR-M plus NIR irradiation (Fig. 7H). The tumor size in the K1-TSHR++ group was significantly smaller than that in the K1-TSHR+ group (Fig. 7I and J), while no further reduction was observed in the K1-TSHR+++ group. These results suggest that a threshold level of TSHR expression is required for effective CAR-mediated targeting and that excessively high expression does not confer additional therapeutic benefit. Given the heterogeneity of TSHR expression in patients, it is important to assess the TSHR expression levels [10]. A limitation of our study is that we did not perform absolute quantification of CAR and TSHR expression levels, nor did we compare these with clinical primary cell samples. Future studies should include such quantifications and comparisons to better understand the relevance of TSHR expression in clinical settings.

## Conclusion

This study developed a genetically engineered cell membrane-coated nanodrug (mPDS@CAR-M), providing an innovative therapeutic strategy for RAIR-DTC. mPDS@CAR-M combines photothermal therapy, chemotherapy, and targeted therapy, effectively inhibiting tumor cell proliferation by enhancing oxidative stress, suppressing the PI3K–AKT–mTOR signaling pathway, and inducing cytotoxic autophagy. Additionally, mPDS@CAR-M activates harmful autophagy to inhibit EMT in tumor cells, thereby reducing their migratory capacity. In vivo experiments demonstrated that mPDS@CAR-M significantly reduced tumor volume, with its therapeutic efficacy being target number dependent. In summary, mPDS@CAR-M exhibits considerable potential in the treatment of RAIR-DTC, and its clinical application warrants further exploration.

## Data Availability

The data supporting the conclusions of this study can be found within the article and its Supplementary Materials files and are also available from the corresponding authors upon reasonable request.
