# Supplementary material for: Genetically Engineered Cell Membrane-Coated Nanodrug for Targeted Treatment of Thyroid Cancer
Source: Biomater Res. 2026 May 7;30:0358. doi: 10.34133/bmr.0358 (PMC13150073; doi:10.34133/bmr.0358)
Supplement: Supplementary 1 — Figs. S1 to S6 Tables S1 to S4 [file bmr.0358.f1.zip › Supplementary Figure.docx]

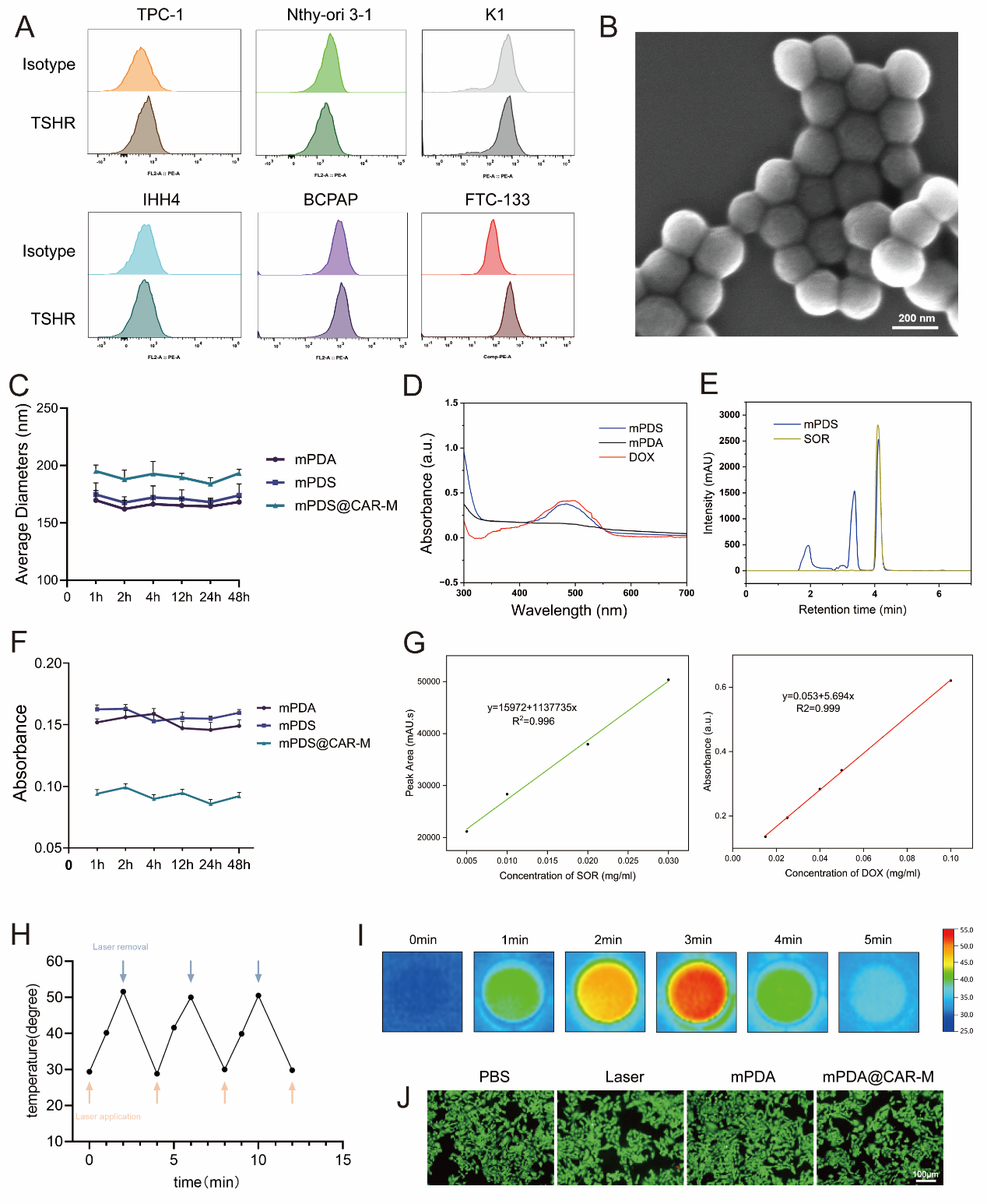


**Supplementary Figure 1**

(A) Flow cytometry showing the expression levels of TSHR in different cell lines. (B) Morphology of mPDS characterized by SEM (scale bar: 200 nm). (C) Particle size stability of mPDA, mPDS, and mPDS@CAR-M over 48 hours. (D) Overlayed UV–Vis spectra of free DOX, mPDA, and mPDS for validation of DOX loading. (E) Representative HPLC chromatograms of free SOR and mPDS confirming SOR encapsulation. (F) Stability of mPDA, mPDS, and mPDS@CAR-M in serum over 48 hours, evaluated by turbidity measurement at 540 nm. (G) Standard curves for the drug concentrations of SOR and DOX. (H, I) Photothermal effect of mPDS@CAR-M under 808 nm NIR irradiation in vitro. (J) Cytotoxicity assays of mPDA, mPDS@CAR-M, and NIR irradiation on cells (scale bar: 100 µm).


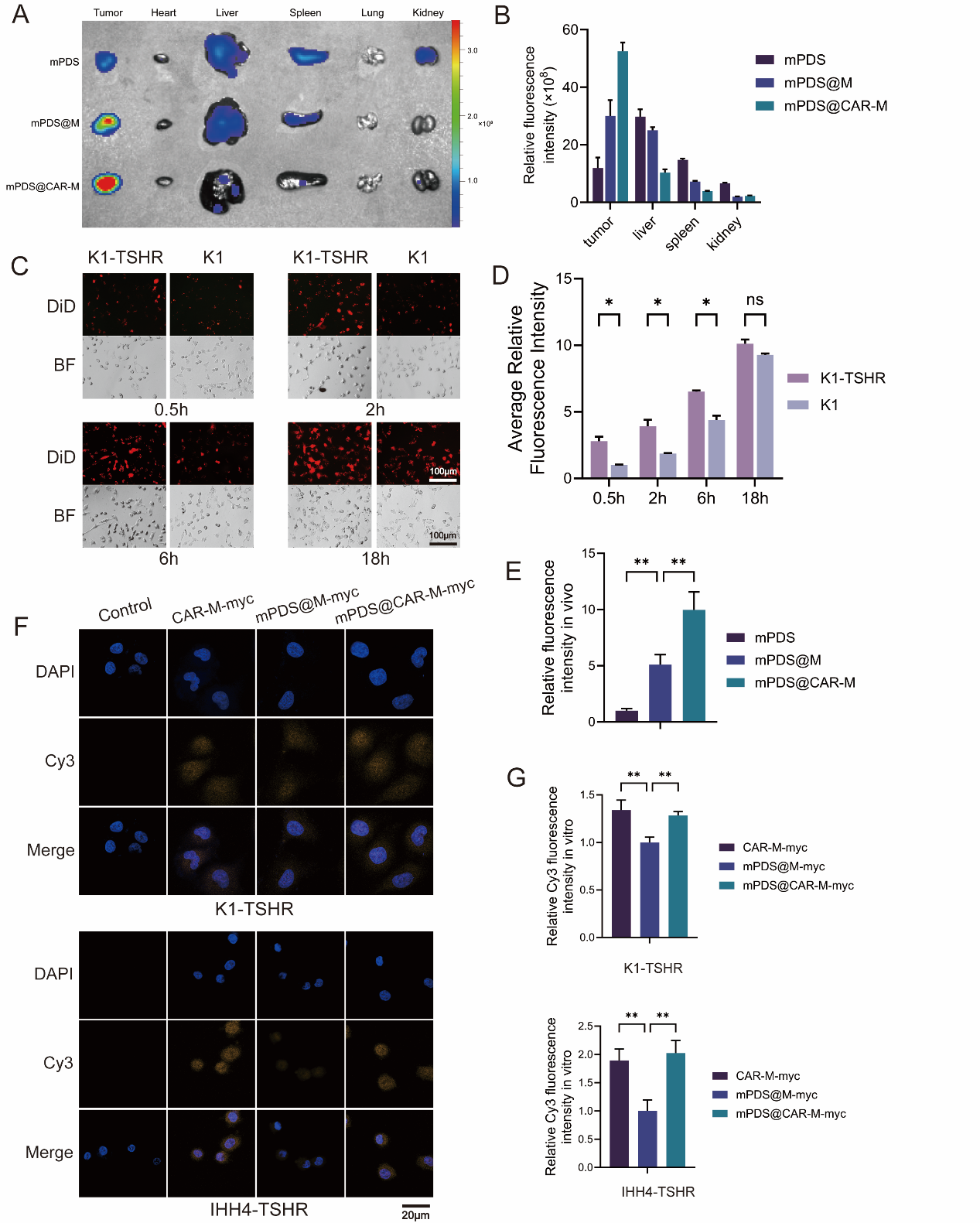


**Supplementary Figure 2**

(A, B) Uptake of mPDS@CAR-M in the heart, liver, spleen, lung, and kidney of different groups, as well as drug distribution in tumors (N = 3). (C) Binding of CAR-M to K1-TSHR cells and K1 cells at different time points (scale bar: 100 μm) and (D) statistical analysis (ns: not significance, *p < 0.05, N = 3). (E) Statistical plot of relative average fluorescence intensity for in vivo targeting. (N = 3; **p < 0.01). (F) Confocal images of Myc/Cy3 fluorescence in K1-TSHR and IHH4-TSHR cells treated with M-myc-or CAR-M-myc-coated nanoparticles. (Scale bar: 20 μm) (G) Quantification of Myc/Cy3 fluorescence intensity in K1-TSHR and IHH4-TSHR cells (N = 3; **p < 0.01).


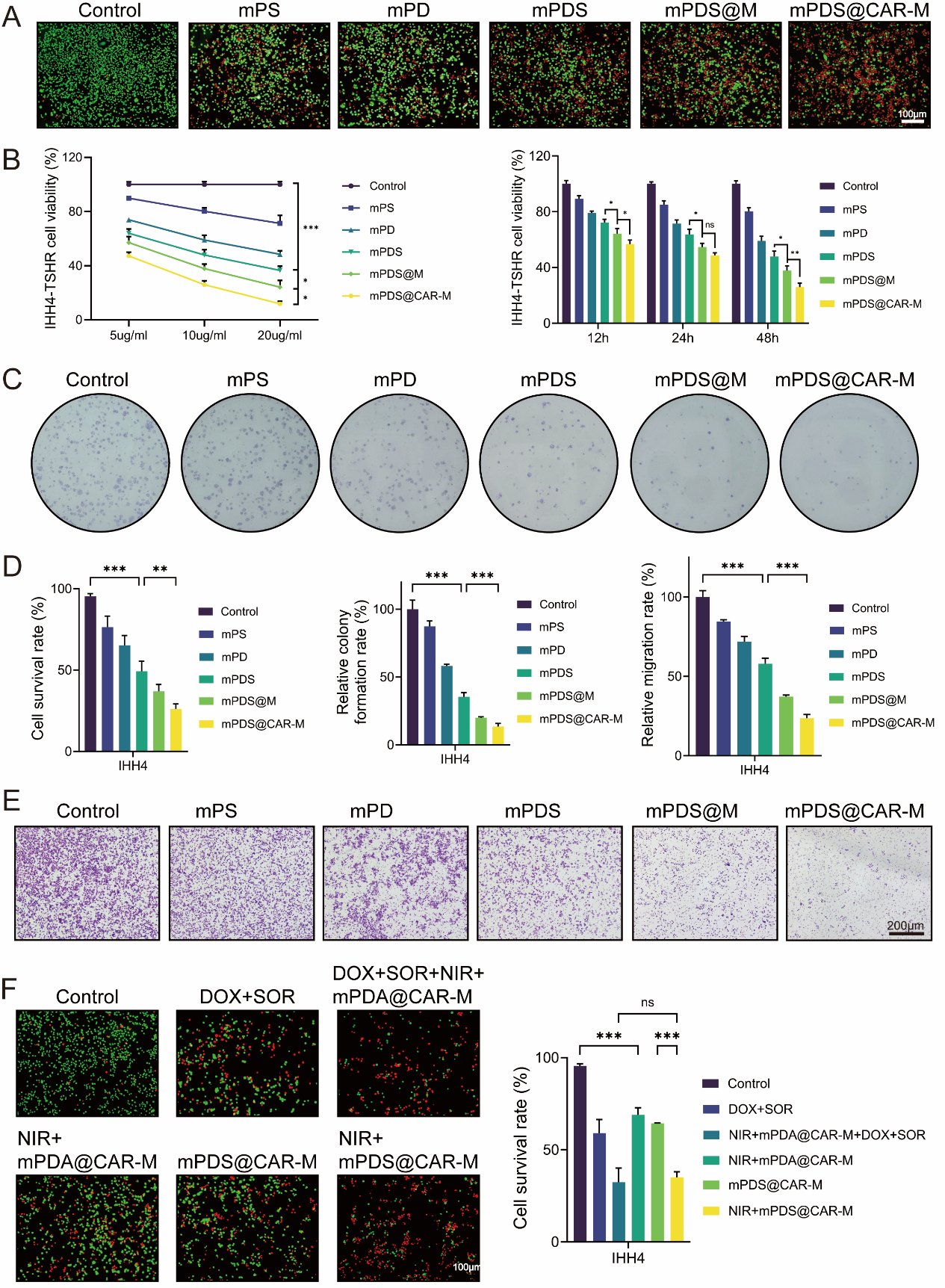


**Supplementary Figure 3**

1. Live/dead staining assay demonstrating the effect of mPDS@CAR-M on IHH4-TSHR cells death (scale bar: 100 μm). (B) CCK-8 assay assessing the effect of mPDS@CAR-M on IHH4-TSHR cells viability (ns: not significance, *p < 0.05, **p < 0.01, ***p < 0.001, N = 3). (C) Colony formation assay showing the impact of mPDS@CAR-M on colony formation in IHH4-TSHR cells. (D) Statistical analysis of live/dead staining, colony formation, and transwell assays (**p < 0.01, ***p < 0.001, N = 3). (E) Transwell assay evaluating the effect of mPDS@CAR-M on IHH4-TSHR cells migration (scale bar: 200 μm). (F) Live/dead staining and quantitative analysis under chemo-photothermal conditions (24 h).


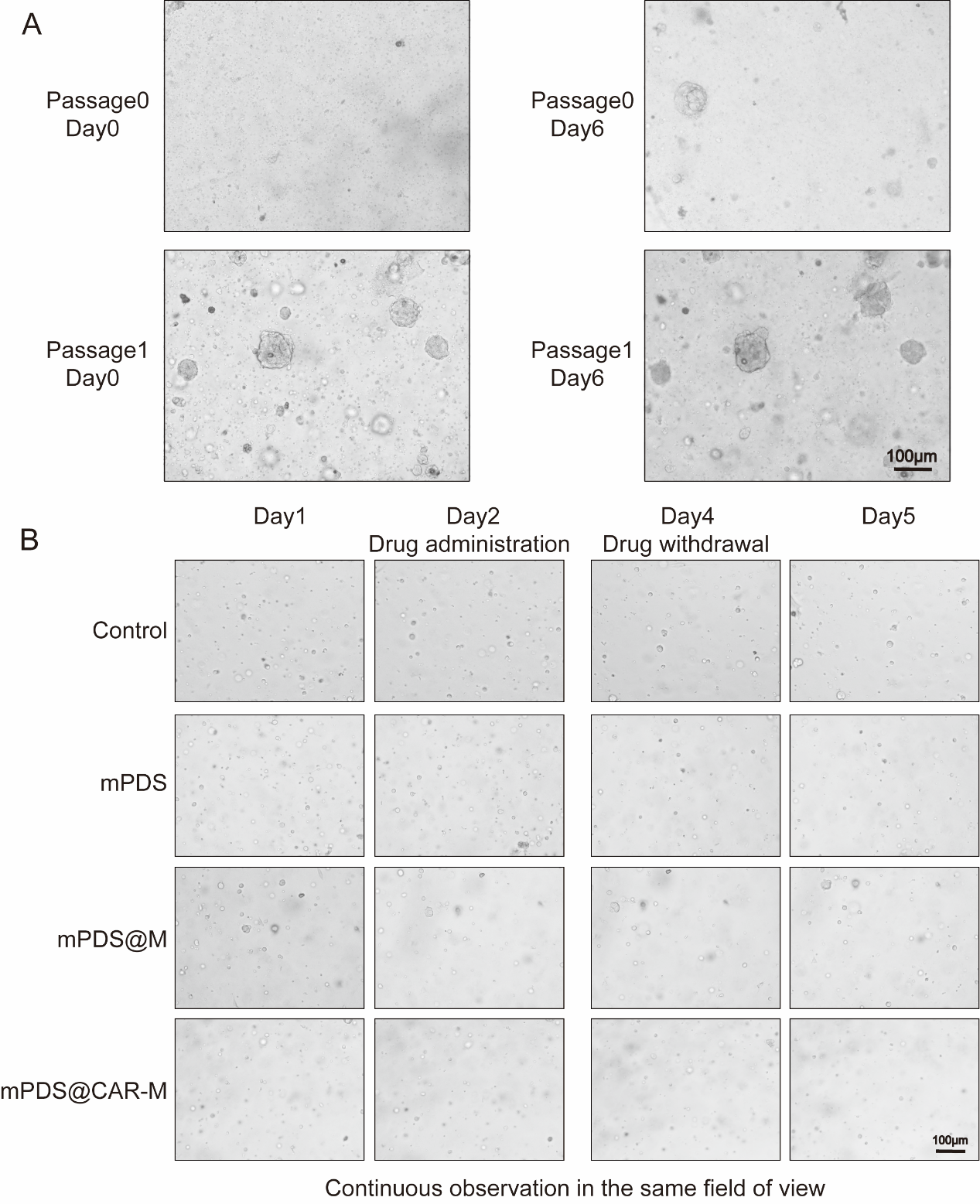


**Supplementary Figure 4**

(A) Primary and passaged organoid culture light microscopy images (scale bar: 100 μm). (B) Light microscopy images of organoids treated with nanodrugs (scale bar: 100 μm).


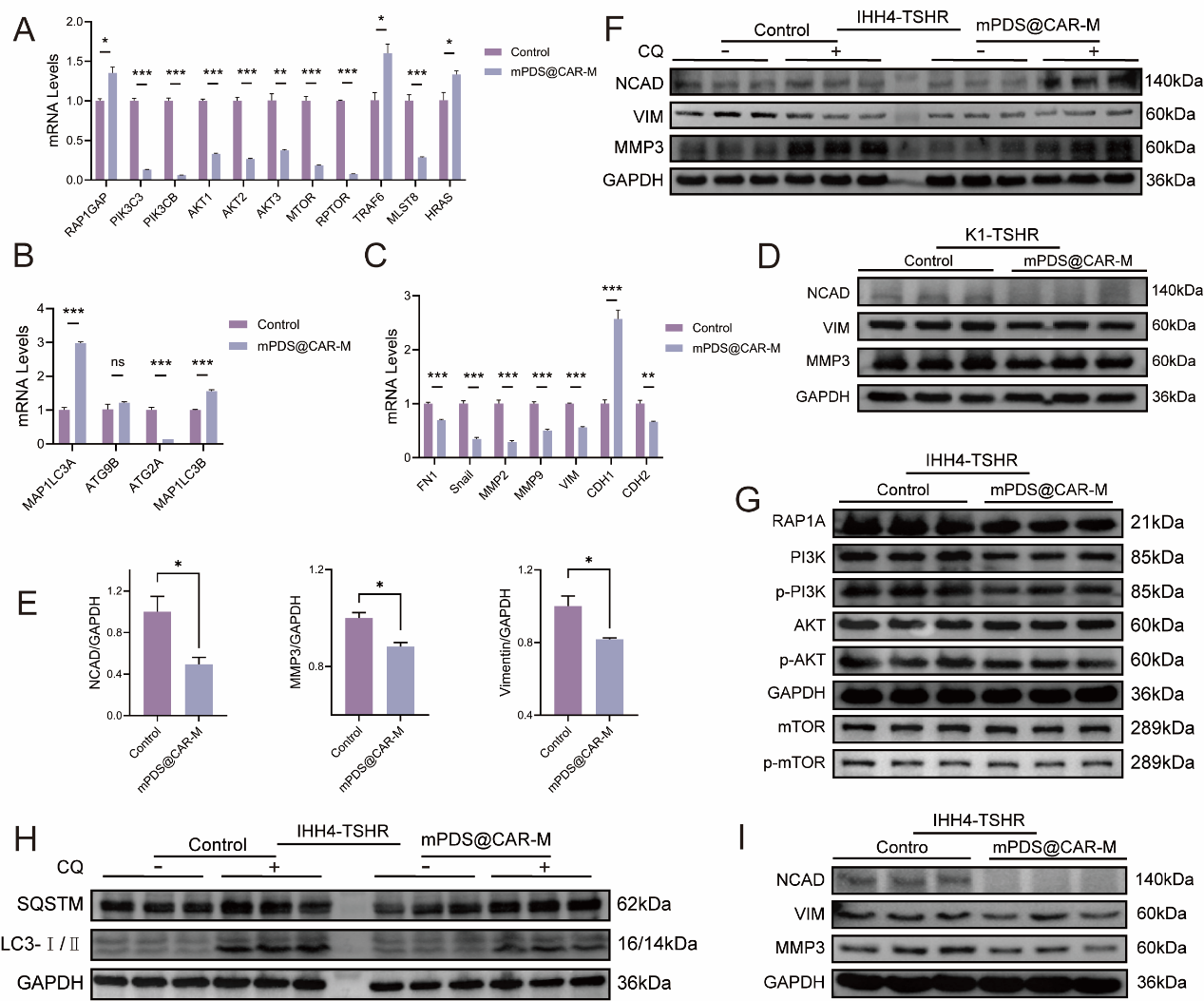


**Supplementary Figure 5**

1. RT-qPCR analysis of the expression of genes related to the RAP1-PI3K-AKT-mTOR signaling pathway in the mPDS@CAR-M group and the control group. (B) RT-qPCR analysis of the expression of genes related to autophagy in the mPDS@CAR-M group and the control group. (C) RT-qPCR analysis of the expression of genes related to EMT in the mPDS@CAR-M group and the control group. (D) Western blot analysis of the expression levels of EMT-related proteins in K1-TSHR cells following mPDS@CAR-M treatment. (E) Quantitative analysis of the proteins in (C) (n = 3, mean ± SD, *p < 0.05). (F) Western blot analysis of EMT-related protein expression in IHH4-TSHR cells after mPDS@CAR-M and CQ treatment. (G) Western blot analysis of key RAP1-PI3K-AKT-mTOR pathway proteins in IHH4-TSHR cells following mPDS@CAR-M treatment. (H) Western blot analysis of autophagy-related protein expression in IHH4-TSHR cells after mPDS@CAR-M and CQ treatment. (I) Western blot analysis of EMT-related protein expression in IHH4-TSHR cells after mPDS@CAR-M and treatment.


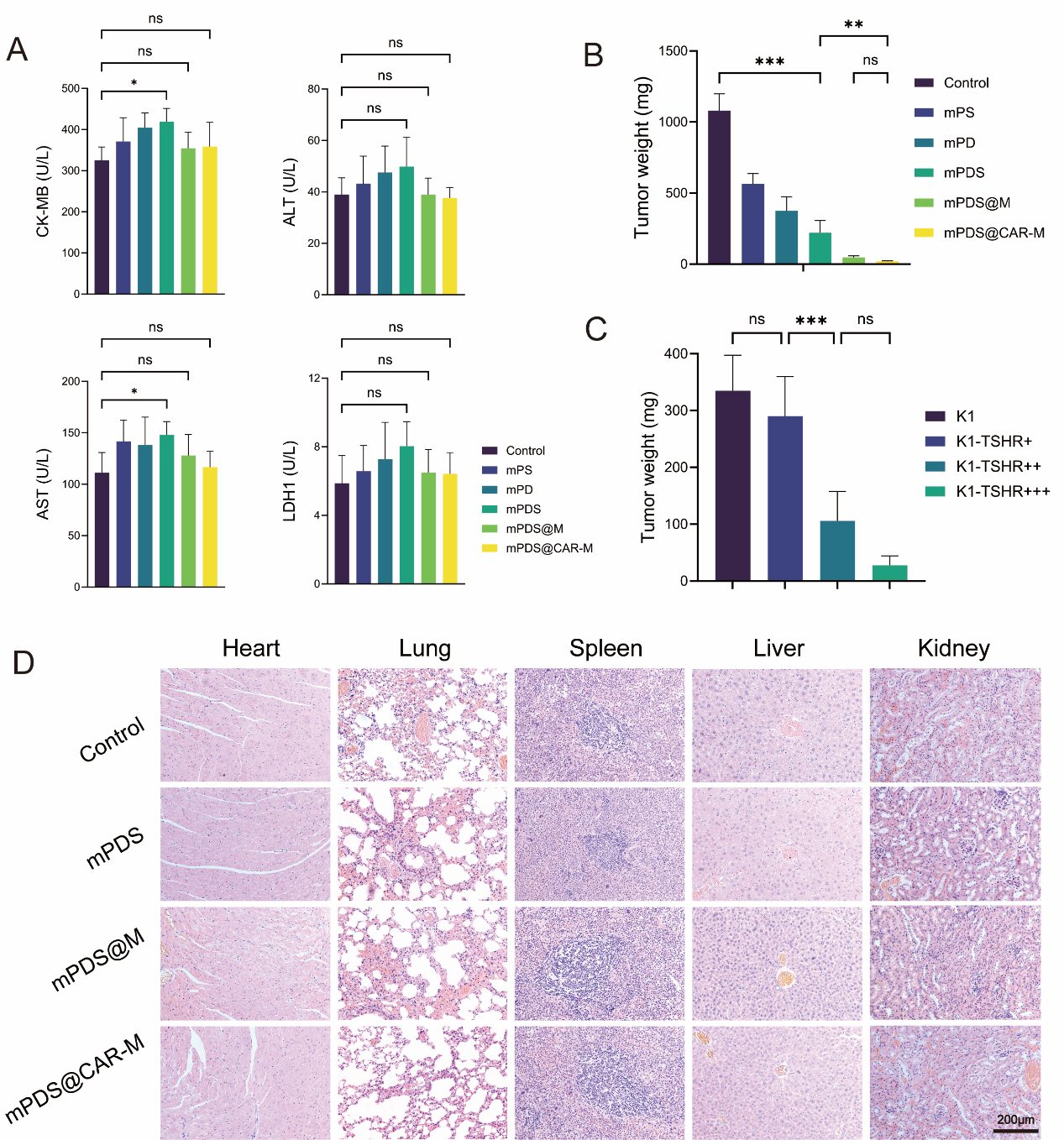


**Supplementary Figure 6**

(A) Quantitative analysis of CK-MB, LDH1, AST, and ALT levels in the blood of nude mice from the control, mPS, mPD, mPDS, mPDS@M, and mPDS@CAR-M groups (ns: not significance, **p < 0.01, N = 6). (B) Final tumor weight of mice from above groups (ns: not significance, **p < 0.01, ***p < 0.001, N = 6). (C) Final tumor weight of K1, K1-TSHR+, K1-TSHR++, and K1-TSHR+++ groups (ns: not significance, **p < 0.01, ***p < 0.001, N = 5). (D) H&E staining of vital organs from tumor-bearing mice in the control, mPDS, mPDS@M, and mPDS@CAR-M groups (scale bar = 200 μm).
